# Phenome-wide screening of the putative causal determinants of bipolar affective disorder using genetic data

**DOI:** 10.1371/journal.pone.0337618

**Published:** 2026-01-02

**Authors:** Bin Hu, Qi Zhou, Hao Wu, Dejiang Yang, Chongyu Xiong, Xiaowei Zhang

**Affiliations:** 1 Department of Clinical Laboratory, The First People’s Hospital of Fuzhou, Fuzhou, Jiangxi, China; 2 Department of Neurology, The First People’s Hospital of Fuzhou, Fuzhou, Jiangxi, China; 3 Department of Neurology, Nanchang First Hospital, Nanchang, Jiangxi, China; 4 Department of Neurology, Jiangxi Provincial People’S Hospital, The First Affiliated Hospital of Nanchang Medical College, Nanchang, Jiangxi Province, China; 5 Department of Neurology, Xiangya Hospital, Central South University, Jiangxi, National Regional Center for Neurological Diseases, Nanchang, Jiangxi Province, China; Institut de Biomedicina de València-CSIC, SPAIN

## Abstract

Bipolar affective disorder (BPAD) is a chronic psychiatric condition with high heritability (60–85%) and significant phenotypic variability, complicating its etiological understanding. Genome-wide association studies (GWAS) have revealed genetic overlaps with diverse traits, suggesting pleiotropy. This study aimed to identify shared genetic factors and infer causal relationships using large-scale GWAS data.BPAD GWAS summary statistics were obtained from the UK Biobank via the Complex Trait Genetics Virtual Lab (CTG-VL) platform. Genetic correlations with 1,504 traits were estimated using linkage disequilibrium score regression (LDSC), with quality filters ensuring heritability (h² > 0.05, p < 0.05). The latent causal variable (LCV) model was applied to assess genetic causal proportion (GCP), distinguishing vertical from horizontal pleiotropy. Multiple testing was corrected using false discovery rate (FDR < 5%).Seventy-five traits showed significant genetic correlations with BPAD (FDR < 5%). Among these, 47 exhibited strong causal associations (|GCP| > 0.6; FDR < 5%), and 28 showed partial genetic causality (|GCP| < 0.6; FDR < 5%). Our results showed that the genetic variants affecting BPAD risk had positive and negative effects on other complex phenotypes. For example, we found that genetic predisposition to BPAD increased the risk of depression, excessive worry, and altered blood levels of vitamin C and calcium. On the other hand, we found that genetic variants influencing the use of certain supplements and medications, such as glucosamine/chondroitin, multivitamin mineral preparations, vitamin D, clopidogrel, and nicorandil, as well as the exposure to gas or solid fuel cooking/heating, decreased BPAD risk. Additionally, we detected that genetic variants affecting some mental, work, living, dietary, and physical health factors also increased BPAD risk. Our study provides novel insights into the potential causal determinants of BPAD and suggests new avenues for future research to elucidate the biological mechanisms and pathways involved in this disorder.

## Introduction

Bipolar affective disorder (BPAD) is a chronic psychiatric condition characterized by recurrent episodes of mania and depression that significantly impair daily functioning [1]. However, there is considerable variability in the clinical presentation of the disorder. This includes differences in the severity and frequency of mood episodes, the presence of comorbidities (e.g., anxiety, substance abuse), and responses to treatment. This variability can complicate the understanding of BPAD’s underlying mechanisms and highlights the need for comprehensive genetic investigations to uncover shared etiological factors.BPAD affects approximately 1% of the global population, imposing a substantial burden through increased risks of suicide, functional impairment, and economic costs [2]. Epidemiological studies underscore its worldwide prevalence, with variations influenced by diagnostic criteria and cultural factors, emphasizing the importance of large-scale datasets for robust analysis.Genetic factors contribute substantially to BPAD susceptibility, with heritability estimates ranging from 60% to 85% based on twin and family studies [3]. These findings suggest a strong polygenic component, where numerous genetic variants of small effect collectively influence risk.

Genome-wide association studies (GWAS) have advanced our knowledge by identifying multiple susceptibility loci for BPAD, revealing overlaps with other psychiatric and non-psychiatric traits [4]. Such discoveries point to pleiotropy, where genetic variants affect multiple phenotypes, necessitating methods to disentangle correlations from causal relationships.Genetic correlations between BPAD and diverse traits, including schizophrenia, intelligence, and metabolic conditions, have been documented, indicating shared genetic architectures [5]. LDSC provides a reliable approach to quantify these correlations using GWAS summary statistics, while controlling for confounding factors like population stratification [6].

To extend beyond correlations and infer causality, the LCV model offers a framework for estimating the GCP, distinguishing vertical pleiotropy from horizontal effects [7]. This method assumes a latent variable mediating causal influences and has been applied to various complex traits.The UK Biobank, a large prospective cohort of European-ancestry individuals, provides rich GWAS data for BPAD and thousands of other phenotypes, enabling phenome-wide analyses [8]. Platforms like the Complex Trait Genetics Virtual Lab (CTG-VL) facilitate access to these resources, incorporating quality filters based on heritability thresholds to ensure analytical reliability [9].

In this study, we leveraged BPAD GWAS summary statistics from the UK Biobank via the CTG-VL platform to estimate genetic correlations with 1,504 traits using LDSC. We further applied the LCV method to assess potential causal associations, aiming to identify novel determinants of BPAD risk and inform its pathophysiology.

## Materials and methods

### BPAD datasets

We obtained the BPAD GWAS summary statistics from the CTG-VL platform [9], which hosts the Neale Lab’s UK Biobank analysis (ICD10 code F31 for bipolar affective disorder). The sample consisted of 387,649 European individuals. Prior to analysis, we verified the SNP-based heritability of BPAD using LD score regression [10].

### CTG-VL datasets

A web-based tool called the CTG-VL platform enables users to access and analyze GWAS data. The platform contains 1,504 GWAS from various sources, mainly from the UK Biobank. The platform filters out GWAS with low or non-significant heritability estimates using LD-score regression, requiring h² > 0.05 and p < 0.05 for inclusion to ensure analytical robustness [9]. The platform also performs downstream analyses, such as clumping, colocalization, and genetic correlation. The GWAS data are from European individuals and are adjusted for confounders such as age, sex, and ancestry. The phenotypes vary from self-reported to measured traits, covering various health and disease outcomes. The platform provides detailed information on each GWAS, as well as links to download or view the data. For our study, we cross-verified heritability estimates for all 1,504 traits via LD score regression outputs from CTG-VL, excluding any that did not meet the criteria prior to genetic correlation analyses.

### Genetic correlations

We estimated the genetic correlations between BPAD and CTG-VL traits using linkage disequilibrium (LD) score regression [10]. LD score regression is a method that uses summary statistics from GWAS to quantify the degree of overlap in genetic variants that influence different traits, while also providing heritability estimates to validate trait suitability. Genetic correlation is a measure of how similar the genetic effects are across traits, ranging from −1 (perfectly negatively correlated) to 1 (perfectly positively correlated). We used the LD Hub web tool [11] to perform LD score regression analysis with default settings, and only proceeded with traits demonstrating significant heritability as noted above. To ensure data quality, we filtered SNPs to include only those from the HapMap 3 reference panel, as these are well-characterized and have high imputation quality [12]. We applied the munge_sumstats.py script from the LDSC repository to format GWAS summary statistics, ensuring allele consistency across datasets by aligning strands and removing ambiguous SNPs (e.g., A/T or C/G SNPs with minor allele frequency > 0.4). Missing data, such as SNPs with incomplete statistics (e.g., missing p-values or effect sizes), were excluded during preprocessing. To address population stratification, we restricted analyses to European-ancestry individuals from the UK Biobank, as principal component analysis (PCA) adjustments for ancestry were applied in the original GWAS data. Additionally, we controlled for confounders such as age and sex, which were adjusted in the CTG-VL platform’s GWAS summary statistics [9].

### Genetic causal proportion

To explore the genetic relationships and causal effects of BPAD with other traits, we performed a large-scale analysis using the CTG-VL platform [9]. We applied LD-score regression and bivariate LCV analysis to assess genetic correlations and vertical pleiotropy (i.e., the influence of a genetic variant on one trait through another trait) among BPAD and 1,463 other phenotypes. We uploaded the BPAD GWAS summary statistics to the CTG-VL and executed the MASSIVE phenome-wide analysis pipeline to estimate genetic correlations and potential causal associations. We visualized the results using causal architecture plots.

The CTG-VL uses R 4.0.0 scripts from the LCV method’s GitHub repository to implement the phenome-wide analysis pipeline [12]. We used the LD score script munge_sumstats.py to format data and ensure consistency of alleles and variants across GWAS summary statistics. We extracted HapMap 3 SNPs using the provided SNP list (w_hm3.snplist) [12]. To assess the causal component of the genetic correlation between two traits, we used a latent variable L that captures the effect of causal variants on both traits [12]. The LCV method computes the GCP as a measure of genetic causality, ranging from −1–1, where zero indicates no causal component (horizontal pleiotropy), and one or −1 indicates a full causal component with no horizontal pleiotropy [12]. A GCP between −0.60 and 0.60 suggests a weak causal component and partial genetic causality [12].

The LCV method assumes that genetic variants have linear effects on traits, that there is no unmeasured confounding beyond population stratification, and that the latent causal variable captures most of the causal effect [12]. Limitations include potential bias from horizontal pleiotropy, where a single variant affects both traits independently, which may inflate causal estimates. Additionally, the method’s accuracy depends on sufficient genetic correlation and heritability, which may limit its power for traits with low heritability. We did not perform sensitivity analyses specifically for BPAD subtype heterogeneity (e.g., bipolar I vs. II) due to the broad ICD10 code F31 definition used in the UK Biobank, which may include mixed subtypes and varying severity levels. To mitigate confounding by population stratification, we relied on the UK Biobank’s PCA-based ancestry adjustments and restricted analyses to European individuals. The LCV model directly addresses horizontal pleiotropy by estimating the extent to which it contributes to the observed genetic correlation, rather than assuming its absence; GCP values near zero highlight cases where horizontal pleiotropy dominates. For population stratification, LCV relies on LDSC, which corrects for this by regressing association statistics against ancestry-matched LD scores from the 1000 Genomes European reference panel, separating true polygenic signals from stratification artifacts. We estimated the genetic correlations of BPAD with 1504 other traits using LD score regression. We then applied the LCV method to test for genetic causality between BPAD and the traits that were genetically correlated. To account for multiple testing, we primarily used the FDR method with a significance threshold of FDR < 5%. This method helps control for the expected proportion of false positives among the multiple comparisons. Additionally, we acknowledge that further corrections, such as the Bonferroni correction, could be applied to ensure even stricter control over false positive findings. Both correction methods were considered in our analysis, with FDR being the main approach to report results in the manuscript, as it balances control of false positives with power in high-dimensional genetic data. We used FDR < 5% to correct for multiple testing and declared significance at adjusted p < 0.05 for the LCV steps.

#### Ethics statement.

This research was conducted using publicly available GWAS summary statistics from the UK Biobank. The UK Biobank study was approved by the North West Multi-centre Research Ethics Committee, and all participants provided written informed consent. Since this study involved only de-identified summary-level data and no direct contact with participants, additional ethical approval and consent were waived.

## Results

Prior to our main analysis, we verified the SNP-based heritability of the BPAD GWAS summary statistics using LD score regression, confirming a significant and substantial heritability estimate (h² = 0.22, SE = 0.0014, p = 4.2e-09) that exceeded standard reliability thresholds (h² > 0.05 and p < 0.05). This established a robust genetic foundation for all subsequent genetic correlation and causal inference analyses.We used the LCV method to explore the genetic relationship and causality between BPAD and 1504 other traits based on GWAS summary statistics. We identified 75 traits that had significant genetic correlation with BPAD (FDR < 5%; S1 Table). Among them, 47 traits showed strong evidence of causal association (|GCP| > 0.6; FDR < 5%; S1 Table) and 28 traits had limited partial genetic causality (|GCP| < 0.6; FDR < 5%; S1 Table).

**Fig 1:**
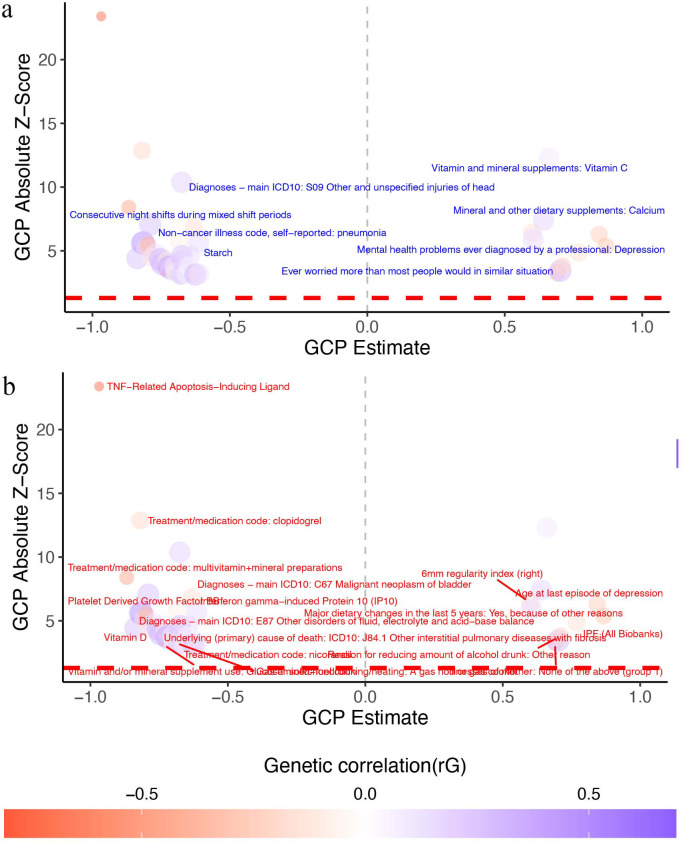
The causal architecture of BPAD. The figure depicts the genetic associations between BPAD and various other phenotypes. Each point represents a trait that has some genetic overlap with BPAD.The horizontal axis shows GCP estimate, and the vertical axis shows GCP absolute Z-score. The red lines indicate the threshold for statistical significance (FDR < 5%). The grey lines indicate the direction of the causal effect: traits on the right increase the risk of BPAD, and traits on the left are increased by BPAD. We divided the traits into two groups: those that have a positive genetic association with BPAD (a) and those that have a negative genetic association with BPAD (b). A GCP estimate of 0 means that there is no direct effect, only a shared genetic background (horizontal pleiotropy). A GCP estimate of 1 or −1 means that there is a complete causal effect. A GCP estimate between −0.60 and 0.60 means that there is a partial causal effect.

Our results revealed that genetic variants influencing BPAD risk had both positive and negative effects on other complex traits (Fig 1 and Table 1). For instance, we found that genetic predisposition to BPAD increased the risk of depression (rG = 0.16, GCP = 0.60, P = 2.31E-07),excessive worry (rG = 0.29, GCP = 0.70, P = 1.40E-02), altered blood levels of Vitamin C (rG = 0.09, GCP = 0.66, P = 1.61E-20) and Calcium (rG = 0.17, GCP = 0.64, P = 1.96E-11).The depression trait analyzed in this study refers to a broad measure of depression, including both unipolar depression and other forms of depressive symptoms, as defined by self-reported diagnosis or clinical records in the UK Biobank. On the other hand, genetic liability for BPAD decreased the risk of illnesses of mother: None of the above (group 1)(rG = −0.27, GCP = 0.69, P = 2.21E-02), age at last episode of depression(rG = −0.25, GCP = 0.84, P = 2.32E-08),reason for reducing amount of alcohol drunk: other reason(rG = −0.22, GCP = 0.71, P = 4.39E-03) and major dietary changes in the last 5 years: yes, because of other reasons(rG = −0.15, GCP = 0.77, P = 4.87E-05).

**Table 1:** Traits influenced by BPAD according to genetic evidence.

Trait	rG	GCP	GCPpval	rGse	rGpval	GCPse	GCP_Z
IPF (All Biobanks)	−0.28	0.87	3.51E-06	0.13	0.85	0.16	5.38
Illnesses of mother: None of the above (group 1)	−0.27	0.69	2.21E-02	0.12	0.82	0.21	3.28
Age at last episode of depression	−0.25	0.84	2.32E-08	0.18	0.94	0.13	6.28
Reason for reducing amount of alcohol drunk: Other reason	−0.22	0.71	4.39E-03	0.11	0.94	0.19	3.79
6mm regularity index (right)	−0.16	0.60	9.79E-09	0.16	0.94	0.09	6.42
Major dietary changes in the last 5 years: Yes, because of other reasons	−0.15	0.77	4.87E-05	0.12	0.94	0.16	4.85
Vitamin and mineral supplements: Vitamin C	0.09	0.66	1.61E-20	0.13	0.96	0.07	12.30
Mental health problems ever diagnosed by a professional: Depression	0.16	0.60	2.31E-07	0.10	0.94	0.10	5.89
Mineral and other dietary supplements: Calcium	0.17	0.64	1.96E-11	0.13	0.94	0.09	7.41
Ever worried more than most people would in similar situation	0.29	0.70	1.40E-02	0.12	0.58	0.20	3.43

Abbreviation: BPAD,bipolar affective disorder.

Trait Trait causally affected by BPAD,GCP Genetic causal proportion,GCP se The standard error of the GCP estimate, GCP pval The p-value of the GCP estimate before FDR correction, rG The coefficient of genetic correlation between the trait and BPAD, rG se The standard error of the rG estimate, rG pval The p-value of the rG estimate before FDR correction.

Moreover, we detected that genetic variants associated with several traits had negative genetic correlations and significant causal proportion with BPAD (Table 2), such as glucosamine/chondroitin(rG = −0.77, GCP = −0.74, P = 2.67E-02), multivitamin mineral preparations(rG = −0.44, GCP = −0.87, P = 1.56E-14), vitamin D (rG = −0.13, GCP = −0.79,P = 2.38E-04), TNF-Related Apoptosis-Inducing Ligand(rG =− −0.70, GCP = −0.97, P = 5.37E-173),Platelet Derived Growth Factor BB (rG = −0.38, GCP = −0.80, P = 1.71E-06),Interferon gamma-induced Protein 10 (IP10)(rG = −0.08, GCP = −0.68, P = 94.21E-07),clopidogrel (rG = −0.20, GCP = −0.82, P = 7.55E-21), nicorandil (rG = −0.18, GCP = −0.63, P = 2.67E-02), and gas or solid-fuel cooking/heating: a gas hob or gas cooker(rG = −0.10, GCP = −0.70, P = 1.87E-02).

**Table 2:** Traits having a negative and significant genetic causal relationship with bipolar affective disorder risk based on FDR < 5% and |GCP| > 0.60 criteria.

Traits	rG	GCP	GCPpval	rGse	rGpval	GCPse	GCP_Z
Vitamin and/or mineral supplement use: Glucosamine/chondroitin	−0.77	−0.74	2.67E-02	0.26	0.31	0.23	3.22
TNF-Related Apoptosis-Inducing Ligand	−0.70	−0.97	5.37E-173	1.08	0.96	0.03	23.39
Treatment/medication code: multivitamin+mineral preparations	−0.44	−0.87	1.56E-14	0.47	0.96	0.10	8.41
Platelet Derived Growth Factor BB	−0.38	−0.80	1.71E-06	0.88	0.97	0.14	5.52
Diagnoses – main ICD10: E87 Other disorders of fluid, electrolyte and acid-base balance	−0.34	−0.81	1.14E-05	0.25	0.94	0.16	5.15
Treatment/medication code: clopidogrel	−0.20	−0.82	7.55E-21	0.20	0.94	0.08	12.86
Treatment/medication code: nicorandil	−0.18	−0.63	2.67E-02	0.28	0.96	0.20	3.21
Diagnoses – main ICD10: C67 Malignant neoplasm of bladder	−0.17	−0.63	8.97E-10	0.25	0.96	0.09	6.84
Vitamin D	−0.13	−0.79	2.38E-04	0.29	0.96	0.17	4.50
Underlying (primary) cause of death: ICD10: J84.1 Other interstitial pulmonary diseases with fibrosis	−0.11	−0.73	7.61E-04	0.22	0.96	0.17	4.23
Gas or solid-fuel cooking/heating: A gas hob or gas cooker	−0.10	−0.70	1.87E-02	0.20	0.96	0.21	3.34
Interferon gamma-induced Protein 10 (IP10)	−0.08	−0.68	4.21E-07	0.26	0.97	0.12	5.79

Abbreviation: BPAD,bipolar affective disorder.

Trait Trait causally affected by BPAD,GCP Genetic causal proportion,GCP se The standard error of the GCP estimate, GCP pval The p-value of the GCP estimate before FDR correction,rG The coefficient of genetic correlation between the trait and BPAD,rG se The standard error of the rG estimate, rG pval The p-value of the rG estimate before FDR correction.

Furthermore, we found that genetic variants influencing several psychiatric-related traits, work environment phenotypes and living and dining environment may increase BPAD risk (Fig 1 and Table 3). Specifically, psychiatric-related traits included diagnosed with life-threatening illness (rG = 0.30, GCP = 0.75, P = 2.35E-03) and ever addicted to any substance or behavior (rG = 0.26,G CP = −0.84,P = 3.40 E −04). Work environment phenotypes included consecutive night shifts during mixed shift periods (rG = 0.26,G CP = −0.79,P = 2.08 E −10), Job SOC coding: Other goods handling and storage occupations N.E.C.(rG = 0.26,G CP = −0.67,P = 1.62 E −04),and current employment status: looking after home and/or family(rG = 0.13,G CP = −0.80,P = 7.40 E −05). living and dining environment included type of accommodation lived in: sheltered accommodation (rG = 0.29,G CP = − 0.63,P = 1.60 E −02) and gas or solid-fuel cooking/heating: none of the above (rG = 0.22,G CP = − 0.67,P = 6.10 E −04).

**Table 3:** Traits having a positive and significant genetic causal relationship with BPAD risk based on FDR < 5% and |GCP| > 0.60 criteria.

Trait	rG	rGse	rGpval	GCP	GCPse	GCPpval	GCP_Z
Diagnoses – main ICD10: I80 Phlebitis and thrombophlebitis	0.05	0.15	0.97	(0.65)	0.17	4.71E-03	3.76
Palmar fascial fibromatosis [Dupuytren]	0.05	0.13	0.97	(0.66)	0.21	2.80E-02	3.19
DVT of lower extremities and pulmonary embolism	0.07	0.13	0.96	(0.67)	0.20	1.39E-02	3.45
Venous thromboembolism	0.08	0.13	0.96	(0.70)	0.18	4.04E-03	3.82
Hair colour (natural, before greying): Black	0.11	0.11	0.94	(0.61)	0.19	2.82E-02	3.18
Other specified/unspecified disorders of synovium and tendon +Other specified/unspecified bursopathies	0.13	0.20	0.96	(0.77)	0.16	6.95E-05	4.78
Current employment status: Looking after home and/or family	0.13	0.19	0.96	(0.80)	0.16	1.74E-05	5.06
Testosterone	0.16	0.07	0.70	(0.65)	0.14	1.66E-04	4.59
Starch	0.16	0.25	0.96	(0.61)	0.11	9.06E-07	5.65
Malignant melanoma of skin	0.18	0.22	0.96	(0.67)	0.20	2.21E-02	3.29
Malignant melanoma of skin	0.18	0.22	0.96	(0.67)	0.20	2.21E-02	3.29
Diagnoses – main ICD10: M13 Other arthritis	0.19	0.28	0.96	(0.68)	0.21	2.80E-02	3.19
Other arthritis (FG)	0.19	0.28	0.96	(0.68)	0.21	2.80E-02	3.19
Diagnoses – main ICD10: S09 Other and unspecified injuries of head	0.20	0.20	0.94	(0.67)	0.07	5.52E-17	10.37
Gas or solid-fuel cooking/heating: None of the above	0.22	0.19	0.94	(0.67)	0.16	7.61E-04	4.23
Diagnoses – main ICD10: K63 Other diseases of intestine	0.23	0.26	0.96	(0.72)	0.20	9.79E-03	3.55
Treatment/medication code: garlic product	0.24	0.32	0.96	(0.74)	0.18	1.23E-03	4.12
Job SOC coding: Other goods handling and storage occupations n.e.c.	0.26	0.41	0.96	(0.67)	0.15	1.62E-04	4.60
Consecutive night shifts during mixed shift periods	0.26	0.33	0.96	(0.79)	0.11	2.08E-10	7.06
Ever addicted to any substance or behaviour	0.26	0.19	0.94	(0.84)	0.19	3.40E-04	4.42
Type of accommodation lived in: Sheltered accommodation	0.29	0.26	0.94	(0.63)	0.20	3.16E-02	3.14
Diagnosed with life-threatening illness	0.30	0.19	0.94	(0.75)	0.19	2.35E-03	3.96
Diagnoses – main ICD10: G37 Other demyelinating diseases of central nervous system	0.31	0.23	0.94	(0.75)	0.17	4.94E-04	4.34
Hypertension	0.37	0.23	0.94	(0.72)	0.19	5.11E-03	3.73
Non-cancer illness code, self-reported: pneumonia	0.48	0.32	0.94	(0.82)	0.15	1.08E-06	5.61

Abbreviation: BPAD,bipolar affective disorder.

Trait Trait causally affected by BPAD,GCP Genetic causal proportion,GCP se The standard error of the GCP estimate, GCP pval The p-value of the GCP estimate before FDR correction,rG The coefficient of genetic correlation between the trait and BPAD, rG se The standard error of the rG estimate, rG pval The p-value of the rG estimate before FDR correction.

Finally, we observed that genetic variations associated with the physical health conditions were shown to increase the BPAD risk. Those diseases included self-reported pneumonia (rG = 0.48, GCP = −0.82, P = 1.08E-06), Hypertension (rG = 0.378, GCP = −0.72, P = 5.11E-03),Other demyelinating diseases of central nervous system(rG = 0.31, GCP = −0.75, P = 4.94E-04),Other diseases of intestine (rG = 0.23, GCP = −0.72, P = 9.79E-03) and Other arthritis(rG = 0.19, GCP = −0.68, P = 2.80E-02).

## Discussion

In this study, we performed a phenome-wide screening of the putative causal determinants of BPAD using genetic data from UK Biobank. We applied a novel method of LDSC with GCP to estimate the genetic relationship and causal proportion between BPAD and 1,436 other traits. We identified 75 genetic correlations with BPAD at FDR < 5%. Of which 47 were inferred to have a causal association (|GCP| > 0.6; FDR < 5%) and 28 showed evidence of limited partial genetic causality (|GCP| < 0.6; FDR < 5%).

Our results confirm previously identified genetic correlations between BPAD and psychiatric conditions such as depression and excessive worry, which are consistent with the well-established comorbidities in BPAD patients. This aligns with findings from genetic studies of depression and anxiety, which have suggested that BPAD shares common genetic risk factors with these disorders, particularly those related to the serotonin transporter gene (SERT) and brain-derived neurotrophic factor (BDNF) [13,14]. The use of the LCV method allows us to estimate not just the genetic correlation but also the causal proportion of these associations, suggesting that the genetic predisposition to BPAD may contribute causally to the increased risk of depression and anxiety. This finding supports the hypothesis that shared biological pathways, such as dysregulated neurotransmission and neuroplasticity, underlie the overlap between BPAD and other psychiatric disorders.

In addition to psychiatric traits, we observed novel genetic correlations between BPAD and immune/metabolic traits such as altered blood levels of vitamin C and calcium, as well as markers related to inflammation (e.g., TNF-Related Apoptosis-Inducing Ligand, Interferon gamma-induced Protein 10). These findings contribute to the growing body of literature that implicates immune dysregulation and inflammation in the pathophysiology of BPAD [15,16]. The genetic associations we identified suggest that individuals genetically predisposed to BPAD may also have altered immune system function, potentially influencing mood regulation through neuroinflammatory pathways. Furthermore, we found that genetic variants affecting BPAD risk were correlated with several physical health conditions, such as hypertension and pneumonia. These correlations suggest that BPAD may share genetic risk factors with conditions that exacerbate inflammation, further supporting the hypothesis that neuroinflammation could be a key driver of BPAD pathophysiology.

We also found genetic correlations between BPAD and various environmental and lifestyle factors, including work-related stress (like consecutive night shifts), living conditions (such as sheltered accommodation), and dietary changes. These are known from prior research to influence BPAD onset and progression, underscoring the role of gene-environment interactions in its etiology. Note that the “heritability” of these environmental traits in GWAS doesn’t mean genes directly control the environment; instead, it reflects gene-environment correlations, where variants shape behaviors, preferences, or socioeconomic factors leading to different exposures [17,18]. For example, links to traits like “gas or solid-fuel cooking/heating” likely stem from broader lifestyle or regional patterns rather than direct environmental causation.A key example is the correlation with consecutive night shifts in mixed schedules, which ties into how shift work disrupts circadian rhythms,a hallmark of BPAD risk. This can affect the suprachiasmatic nucleus, the brain’s main clock, causing erratic melatonin levels and imbalances in serotonin and dopamine systems essential for mood control [19]. Long-term studies show that ongoing circadian disruption in shift workers raises mood episode risks through HPA axis overdrive, higher cortisol, neuroinflammation, and impaired hippocampal neurogenesis, all tied to BPAD [20]. Our data hint that shared variants may involve clock genes like CLOCK and PER3, which govern sleep and link to manic-depressive patterns [21]. This points to possible preventive steps, like light therapy or melatonin supplements, for those at genetic risk.Another factor is sheltered accommodation, which showed a positive genetic link to BPAD risk. Studies back this, revealing higher bipolar I rates in sheltered settings versus the general population, often due to socioeconomic challenges, social disconnection, and limited mental health access [22]. In one older adult cohort, bipolar cases in sheltered housing had more impairment and comorbidities, indicating these settings may heighten risks for genetically vulnerable people [22]. On a biological level, chronic stressors like isolation could dysregulate the HPA axis, spiking cortisol and throwing off circadian and neurotransmitter functions, fueling BPAD episodes [23]. Overall, this highlights how environment and genetics interact in BPAD.Future work should delve deeper into these interactions to clarify their impact on BPAD development.

In our analysis, we found several traits with high GCP that require further exploration. For instance, the genetic predisposition to multivitamin use was associated with a reduced BPAD risk. This could be explained by the well-documented role of vitamins, particularly B-vitamins, in neural health and the reduction of oxidative stress, both of which are important in the pathophysiology of BPAD. Studies have shown that multivitamin supplementation can positively affect mood regulation and neuronal function, which may provide a protective effect for those genetically predisposed to BPAD [24].Similarly, clopidogrel, an antiplatelet medication, was found to have a negative genetic correlation with BPAD risk. The potential mechanism behind this could involve clopidogrel’s anti-inflammatory properties. Inflammation has been implicated in the development and progression of BPAD, and medications that target inflammation could therefore offer a therapeutic avenue for individuals at genetic risk for the disorder. The role of anti-inflammatory drugs in mental health has gained increasing attention, with studies suggesting that reducing inflammatory markers may mitigate mood disorder symptoms [25].Furthermore, we observed that genetic variants associated with exposure to gas were correlated with an increased BPAD risk. This finding warrants further investigation into how environmental exposures contribute to psychiatric disorders. Such exposures could lead to chronic inflammation or respiratory conditions, which are known to be associated with an increased risk of mood disorders. The interaction between genetic susceptibility and environmental factors like gas exposure could offer a new understanding of BPAD etiology and inform preventive strategies [26].

We also observed that genetic liability for several physical health conditions, such as hypertension, pneumonia, demyelinating diseases, intestinal diseases, and arthritis, may increase the risk of BPAD. These results are in line with the epidemiological evidence for the high prevalence and morbidity of medical comorbidities in patients with BPAD [27]. The co-occurrence of physical and mental disorders may be explained by several factors, such as shared genetic risk factors, environmental exposures, lifestyle factors, medication effects, or bidirectional causal relationships. For example, hypertension may increase the risk of BPAD by affecting cerebral blood flow and brain function [28], while pneumonia may trigger or exacerbate mood episodes by inducing systemic inflammation and cytokine release [29].

The use of the LCV method, a form of Mendelian randomization, allows us to infer causality between BPAD and other traits. By utilizing genetic variants as instrumental variables, we can more accurately estimate the direction of causality, distinguishing between correlation and causation. Our results suggest that BPAD risk is not only genetically correlated with other complex traits but also has a causal influence on certain conditions, such as depression and altered blood levels of vitamin C. This approach strengthens existing hypotheses about the role of shared biological mechanisms in BPAD and its comorbidities and offers a more precise understanding of the genetic underpinnings of BPAD. The LCV method helps to clarify the causal relationships and supports the notion that BPAD may contribute to the onset of other disorders rather than merely co-occurring with them.

Our study has several strengths. First, we utilized a large sample size from the UK Biobank, which enhanced our statistical power to detect genetic correlations and causality between BPAD and other traits. Second, we employed a novel method combining LDSC with GCP, enabling us to estimate both the magnitude and direction of causality between traits [30].However, our study also has limitations. One key limitation is that the results may be subject to confounding by population stratification, pleiotropy, or horizontal pleiotropy, particularly for environmental traits where gene-environment correlations could mimic direct effects [31]. Therefore, our findings should be interpreted with caution and validated by independent methods, such as Mendelian randomization or bidirectional polygenic risk score analysis. Another limitation is that we used a broad definition of BPAD based on self-reported diagnosis, health records, or registry data, which may have introduced phenotypic heterogeneity and misclassification bias. This heterogeneity,encompassing variations in subtypes (e.g., bipolar I vs. II), severity, or episode patterns,could dilute specific genetic signals, potentially leading to underestimated genetic correlations for traits linked more strongly to certain subtypes or biased causal inferences if unaccounted pleiotropic effects differ across subgroups [32]. For example, variants associated with rapid-cycling BPAD might show distinct correlations compared to those in less severe forms, affecting the reliability of GCP estimates. This hybrid approach lacks direct psychometric validation against gold-standard structured or semi-structured interviews, which are known to offer higher diagnostic reliability for BPAD in epidemiological studies. Additionally, our analysis relied exclusively on data from European-ancestry individuals, which limits the applicability of our findings to more diverse populations and may overlook ancestry-specific genetic effects or interactions. This is particularly relevant given the global prevalence of bipolar spectrum disorders across varied ethnic groups [1], and the potential for ancestry bias in GWAS to exacerbate health disparities.Although the CTG-VL platform primarily hosts European-ancestry GWAS, we did not test non-European datasets due to their limited availability within the platform for our specific analyses; future studies could address this by incorporating emerging multi-ancestry resources. Furthermore, although we identified 75 genetic correlations with BPAD at FDR < 5% and applied a conservative FDR threshold to minimize false positives, some of these associations may still be false positives [33]. The use of complementary methods such as LDSC and GCP helps to mitigate this risk, but we recommend independent replication of these findings in additional cohorts to validate these associations and explore their robustness further.

## Conclusions

In conclusion, our study provides a comprehensive and systematic analysis of the genetic determinants of BPAD using phenome-wide data from UK Biobank. We identified novel genetic correlations and causal associations between BPAD and other traits, which shed new light on the biological origins and potential mechanisms of BPAD. Our findings also suggest some promising genes and pathways for further research on the pathophysiology and treatment of BPAD. Moreover, our study demonstrates the utility and feasibility of applying LDSC with GCP to explore the genetic architecture and causality of complex traits.

## Supporting information

S1 Table LCV analysis for bipolar affective disorder. (XLSX)
